# Genetic and cellular studies highlight that A Disintegrin and Metalloproteinase 19 is a protective biomarker in human prostate cancer

**DOI:** 10.1186/s12885-016-2178-4

**Published:** 2016-02-24

**Authors:** Gerard Hoyne, Caroline Rudnicka, Qing-Xiang Sang, Mark Roycik, Sarah Howarth, Peter Leedman, Markus Schlaich, Patrick Candy, Vance Matthews

**Affiliations:** School of Health Sciences and Institute of Health Science Research, The University of Notre Dame Australia, Fremantle Campus, Australia; Royal Perth Hospital, Perth, Australia; Department of Chemistry and Biochemistry, Florida State University, Tallahassee, Florida USA; Harry Perkins Institute of Medical Research and the Centre for Medical Research, The University of Western Australia, Perth, Australia; School of Medicine and Pharmacology - Royal Perth Hospital Unit, The University of Western Australia, Perth, Australia; School of Medicine and Pharmacology- Royal Perth Hospital Unit, Level 3, Medical Research Foundation Building, Rear 50 Murray Street, Perth, WA 6000 Australia

**Keywords:** ADAM19, Prostate cancer, Proliferation, Metalloproteinase, Microarray

## Abstract

**Background:**

Prostate cancer is the second most frequently diagnosed cancer in men worldwide. Current treatments include surgery, androgen ablation and radiation. Introduction of more targeted therapies in prostate cancer, based on a detailed knowledge of the signalling pathways, aims to reduce side effects, leading to better clinical outcomes for the patient. ADAM19 (A Disintegrin And Metalloproteinase 19) is a transmembrane and soluble protein which can regulate cell phenotype through cell adhesion and proteolysis. ADAM19 has been positively associated with numerous diseases, but has not been shown to be a tumor suppressor in the pathogenesis of any human cancers. Our group sought to investigate the role of ADAM19 in human prostate cancer.

**Methods:**

ADAM19 mRNA and protein levels were assessed in well characterised human prostate cancer cohorts. ADAM19 expression was assessed in normal prostate epithelial cells (RWPE-1) and prostate cancer cells (LNCaP, PC3) using western blotting and immunocytochemistry. Proliferation assays were conducted in LNCaP cells in which ADAM19 was over-expressed. In vitro scratch assays were performed in PC3 cells over-expressing ADAM19.

**Results:**

Immunohistochemical studies highlighted that ADAM19 protein levels were elevated in normal prostate tissue compared to prostate cancer biopsies. Results from the clinical cohorts demonstrated that high levels of ADAM19 in microarrays are positively associated with lower stage (*p* = 0.02591) and reduced relapse (*p* = 0.00277) of human prostate cancer. In vitro, ADAM19 expression was higher in RWPE-1 cells compared to LNCaP cells. In addition, human ADAM19 over-expression reduced LNCaP cell proliferation and PC3 cell migration.

**Conclusions:**

Taken together, our immunohistochemical and microarray results and cellular studies have shown for the first time that ADAM19 is a protective factor for human prostate cancer. Further, this study suggests that upregulation of ADAM19 expression could be of therapeutic potential in human prostate cancer.

**Electronic supplementary material:**

The online version of this article (doi:10.1186/s12885-016-2178-4) contains supplementary material, which is available to authorized users.

## Background

Recent estimates suggest that 1.1 million cases of prostate cancer were diagnosed worldwide [1]. Prostate cancer is the second most common cancer in men, and the fifth most common cause of cancer-related deaths in men [1]. The age-adjusted incidence of prostate cancer has risen in line with an increase in the number of men being tested and improvements in widespread diagnostic testing [1].

Early-stage prostate cancer tumours require androgens as growth factors for proliferation and survival [2]. Androgen deprivation may be successfully implemented to treat androgen-dependent prostate cancer tissue, but is ineffective at treating androgen-independent prostate cancer tissue [3]. Androgen ablation therapy also impacts the growth and survival of normal prostate epithelium [2] and has an undesirable effect on body composition and other physiological and metabolic parameters, thus increasing the risks for other diseases, such as osteoporosis [4]. Increased specificity of treatment reduces the risk of these side effects and is more likely to result in long term decreases in proliferation and metastasis of cancer, leading to improved clinical outcomes [5]. It is therefore important to further develop treatment options which specifically target prostate cancer cells [5].

Numerous mechanisms underlying the pathogenesis of prostate cancer have been identified. For example, enhanced levels of the mitogen insulin-like growth factor 1 (IGF-1) and low levels of IGFBP-3 are associated with a higher risk of prostate cancer [6]. Previous studies have also indicated that inhibition of the IGF-1 receptor reduced invasive activity of PC-3 human prostate cancer cells [7]. There is still a real need to understand novel mechanisms that underlie prostate cancer pathogenesis.

Metalloproteinases, or ADAM proteins (A Disintegrin And Metalloproteinase), are proteolytic enzymes that are linked with the malignant progression of human prostate cancer [8]. ADAMs are a family of transmembrane and secreted proteins which regulate cell phenotype through affecting cell adhesion, migration, proteolysis and signalling [8]. Twenty-one human ADAMs have been described and many have been positively associated with the pathogenesis of human prostate cancer. ADAM9 expression is significantly higher in prostate cancer tissue than normal prostate tissue [9] and inhibition of ADAM9 expression in prostate cancer enhanced prostate cancer sensitivity to radiation and chemotherapy [10]. Knockdown of ADAM10 decreased proliferation of prostate cancer cells, suggesting that ADAM10 may contribute to the progression of prostate cancer by increasing proliferation [11]. ADAM15 has been shown to contribute to the metastatic progression of human prostate cancer through the binding of its disintegrin domain to various integrins [12]. Finally, Xiao et al*.* [13] showed that ADAM 17 increased the invasive capacity of prostate cancer cells by targeting matrix metalloproteinases (MMPs) two and nine.

ADAM19, also known as meltrin β, was identified and characterised by our team [14, 15] and others [16]. ADAM19 has been linked to numerous diseases [14] and serves important biological functions in embryogenesis [17], cardiovascular system development [18] and in skeletal muscle adaptation [19]. ADAM19 contains several domains, including a prodomain, metalloproteinase domain, disintegrin domain, cysteine-rich domain, epidermal growth factor-like domain, transmembrane domain and cytoplasmic tail domain [8]. The metalloproteinase domain of ADAM19 is known to be involved in extracellular matrix breakdown and reconstruction [15]. One of the most important functions carried out by the metalloproteinase domain of ADAM19 is the catalytically-mediated ectodomain shedding of substrates [15]. The disintegrin domain of ADAM19 functions as an adhesion domain by binding to integrins α4β1 and α5β1 and inhibiting their function [20]. Importantly, both of these integrins have been implicated in the development of cancer metastases, including that of prostate cancer [21].

Based on the emerging evidence of ADAM involvement in human cancer, we were interested to investigate if ADAM19 might play a role in prostate cancer using a combination of clinical cohorts and in vitro analyses. We found that ADAM19 is a tumor suppressor in human prostate cancer patients and that it inhibits prostate cancer cell proliferation and migration in cell culture.

## Methods

### ADAM19 immunohistochemistry

ADAM19 immunohistochemistry was conducted on human prostate cancer samples contained on the Prostate Cancer Tissue Array (Abcam, #ab178263). We personally did not have to gain ethics approval as samples were part of a commercially available tissue array. All tissue was examined/diagnosed by a licensed pathologist and was ethically obtained. Immunohistochemistry was conducted using standard procedures with primary antibody (rabbit anti-hADAM19 disintegrin domain IgG (pAb362)) at a 1:200 dilution [22, 23].

### Secondary analysis of gene expression omnibus (GEO) gene expression microarray data

A human prostate cancer microarray of 71 patients (GEO accession number: GSE40272) contained information on ADAM19 gene expression in human prostate tumours, and was processed using the R ‘affy’ and ‘limma’ packages. In addition, we investigated the clinical significance of human ADAM19 expression in human prostate cancer tumour tissue in this cohort of patients, as follow up clinical data was available.

We also analysed intratumoural RNA-seq expression data from a cohort of 156 patients with prostate cancer available at The Cancer Genome Atlas (TCGA) (http://tcga-data.nci.nih.gov/tcga/tcgaDownload.jsp); accessed June 2013). This cohort consisted of 65 patients with pathologically determined stage II prostate cancer, 85 patients with stage III, 5 patients with stage IV prostate cancer and one patient of unknown staging. The mean age of patients in this cohort was 60.3 years.

We personally did not have to gain ethics approval as analysis was performed on publicly available microarray data. The Cancer Genome Atlas (TCGA) is advised by an External Scientific Committee whose membership includes patient advocates, senior scientists and clinicians with relevant expertise in ethics. All prostate samples used in the GSE40272 related study were collected with patient’s informed consent under an Institutional Review Board approved protocol.

### Cell culture experiments

Normal human epithelial prostate cells (RWPE-1), which express the androgen receptor, were compared with androgen sensitive, human prostate cancer cells (LNCaP). Androgen independent human prostate cancer cells (PC3) were used for in vitro scratch assays due to their ability to produce a monolayer in culture. Human embryonic kidney cells (HEK293) were used for tumor necrosis factor-α (TNF-α) shedding experiments.

All cells were purchased from the American Type Culture Collection (Manassas, VA, USA). HEK293 cells were cultured in Dulbecco’s Modified Eagle Medium (DMEM) [low glucose; Gibco] containing 10 % fetal calf serum (FCS) and 1 % penicillin/streptomycin (Invitrogen, USA). RWPE-1 cells were cultured in Keratinocyte Serum Free Medium (K-SFM; GIBCO) containing 0.05 mg/ml bovine pituitary extract (BPE) and 5 ng/ml human recombinant epidermal growth factor (EGF) provided with the K-SFM kit. LNCaP and PC3 cells were cultured in Roswell Park Memorial Institute-1640 media (RPMI) (Sigma-Aldrich, Germany) with 10 % FCS and 1 % Penicillin/Streptomycin. To maintain viable healthy and undifferentiated cells, RWPE-1, LNCaP and PC3 cells were maintained until they reached 70 % confluency and were then transferred into a 75 cm^2^ flask. Cells were split into 6 well Cell Bind (Costar), 12 well Cell Bind (Costar) or 96 well cell culture plates for further studies.

### Determination of protein expression

LNCaP and RWPE-1 cells were harvested and washed with cold 1X PBS. Cells were lysed using cytosolic extraction buffer (10 mM hydroxyethyl piperazineethanesulfonic acid; 3 mM MgCl_2_; 14 mM KCl; 5 % glycerol; 0.2 % IGEPAL) containing phosphatase and protease inhibitors (Roche). Cells were then scraped and lysates were transferred to a 1.5 mL eppendorf tube and stored at -80 °C. After 24 h, lysates were centrifuged at 13 000 rpm at 4 **°**C for 10 min. Bradford assay (Bio-Rad, Hercules, CA, USA) was used to determine protein concentrations. Protein lysates (40 μg) were solubilized in Laemmeli sample buffer and boiled for 10 min, resolved by sodium dodecyl sulfate (SDS)–polyacrylamide gel electrophoresis on 10 % polyacrylamide gels, transferred by semi-dry transfer to polyvinylidene difluoride membrane and blocked with 5 % milk powder. Membranes were then incubated overnight at 4 °C in primary antibodies [rabbit anti-hADAM19 metalloproteinase domain IgG (pAb361) [22] or mouse anti-β-actin (Abcam, Cambridge, UK; ab6276)] using recommended dilutions. Membranes were washed three times in washing buffer and incubated for 60 min at room temperature with either anti-rabbit or anti-mouse horse-radish peroxidase (HRP; Sigma, USA) respectively. Membranes were then washed and briefly incubated in Amersham ECL Prime Western Blotting Detection Reagent (GE). The protein bands were detected using the Alpha Innotech MultiImage II Fluor Chem FC2.

### Cell transfections

Transfections were conducted in 6 or 12 well Cell Bind (Costar) culture plates. Transfection was carried out once adherent HEK293 or PC-3 cells reached approximately 70 % confluency using Lipofectamine™ 2000 (Invitrogen, Calsbad, California, USA). Alternatively, LNCaP cells were transfected in suspension. Cells were transfected with either pcDNA3.1 GFP vector (Invitrogen), empty pCR3.1 vector [23] or vectors containing the cDNA for human ADAM19 (pCR3.1 hADAM19) [23] or human TNF-α (pcDNA3.1 (-) pro-TNF-α) [24]. Cells were incubated at 37 °C, in 95 % O_2_/5 % CO_2_. Cells were visualised for GFP using the Nikon Eclipse Ti microscope to evaluate transfection efficiency after 24 and 48 h. Cell-free culture supernatants were collected after 48 h. Transfected cells were then used for immunocytochemistry to evaluate ADAM19 expression. In addition, ADAM19 transfected cells were used in MTS proliferation assays or migration studies. Empty vector-transfected cells were used as a comparative control.

### Immunocytochemistry

Immunocytochemistry was used to confirm basal level and over-expression of human ADAM19 in LNCaP, RWPE-1, PC-3 and HEK293 cell lines. Cells were fixed in methanol/acetone (1:1) and endogenous peroxidases blocked using 0.3 % hydrogen peroxide in Triton X/PBS (Tx/PBS) for 5 min. Cells were blocked for 1 h in 10 % FCS/Tx/PBS, incubated with primary antibody (rabbit anti-hADAM19 disintegrin domain IgG (pAb362) [23]) at 4 °C overnight, washed 3X in Tx/PBS for 5 min before a secondary antibody [anti-rabbit horse-radish peroxidase (HRP) (Sigma, USA) diluted 1:100 in blocking buffer (10 % FCS/Tx/PBS)] was added for 45 min. Cells were washed 2X in Tx/PBS for 5 min before diaminobenzidine (DAB, DAKO) was added for approximately 10 min and cells were then visualised. Negative controls had the primary antibody omitted which resulted in no staining. Cells were visualised using the Nikon Eclipse Ti microscope.

### MTS assay

Transfected and untransfected LNCaP cells were resuspended at 0.25x10^5^ cells/mL in RPMI-1640 medium containing 10 % FCS and 1 % streptomycin/ penicillin and added in 100 μl volumes to the centre of the wells of a 96-well culture plate. This technique allowed even dispersion of cells in the well. We plated 12 samples per cell type per treatment per time point. After 1, 3, 5 and 7 days, the medium was carefully aspirated and 100 μl RPMI-1640 medium containing 10 % FCS and 1 % streptomycin penicillin containing 20 μl of MTS assay reagent was added to each well for 3 h. After incubation at 37 **°**C, in 95 % O_2_/5 % CO_2_, proliferation was determined by MTS assay. Plates were read at 490 nm (0.1 s per well) on a plate reader. The Nikon Eclipse Ti microscope was used at each required time point to image cells.

### TNF-α ELISA

Human TNF-α in the cell-free culture supernatant collected from transfected HEK293 or PC3 cells was measured using a commercially available enzyme-linked immunosorbent assay kit (TNF-α; R&D Systems, DY210).

### In vitro scratch assay

Migration of transfected PC3 cells was assessed using an in vitro scratch assay [25]. Cell death was determined with trypan blue cell counting.

### Statistics

Statistical analysis of microarrays was performed using the R programming environment. The Kaplan-Meier survival curve was based on unadjusted Cox regression of GSE40272 data using the R “survival” package. The median was used to divide tumours into high or low intratumoral ADAM19 expressing groups for comparison with longitudinal survival.

The TCGA boxplot was produced using the R “graphics” package and showed the correlation between tumour stage and human ADAM19 expression. This data was additionally assessed using Pearson’s product moment correlation. Statistician Dr. Patrick Candy performed the microarray statistical analysis (Harry Perkins Institute of Medical Research, University of Western Australia, Australia).

In the cell culture experiments, all data was analysed from three independent experiments and data was statistically analysed using paired t-tests where appropriate. Statistical significance was determined if the probability of the null hypothesis was less than 0.05 (*p* ≤ 0.05). GraphPad Prism6 was used to plot the data (GraphPad Software, Inc., LaJolla, CA).

## Results

### Human prostate carcinoma tissue displays lower ADAM19 expression

**Fig. 1:**
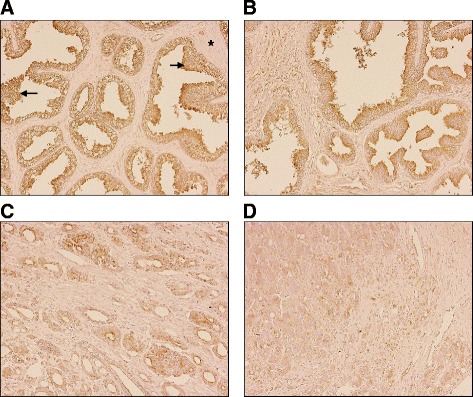
Immunostaining of ADAM19 and its correlation with severity in human prostate cancer**.** ADAM19 immunostaining of (**a**) normal prostate, exhibiting hyperplasia; (**b**) prostate hyperplasia; (**c**) malignant prostate adenocarcinoma, grade II; and (**d**) malignant prostate adenocarcinoma, grade III. Photomicrographs are 200X magnification. Asterisk indicates stroma and arrow indicates glandular hyperplasia. ADAM19 staining is brown in colour. Haematoxylin counterstaining is purple in colour

Human prostate tumour biopsies and normal prostate tissue samples on a Prostate Cancer Tissue Array were immunostained for ADAM19. In normal human prostate tissue, ADAM19 is highly expressed on the luminal surface of glandular epithelial cells as indicated by brown diaminobenzidene staining (Fig. 1a). Excitingly, we report for the first time that human prostate carcinoma samples have low ADAM19 expression (Fig. 1c and d) when compared with benign prostate hyperplasia samples (BPH; Fig. 1a and b). Intriguingly, human ADAM19 expression is reduced as the severity of prostate cancer rises (Fig. 1c and d) which is a novel finding.

### High ADAM19 expression correlates with increased disease-free survival from prostate cancer, and lower tumour stage

**Fig. 2:**
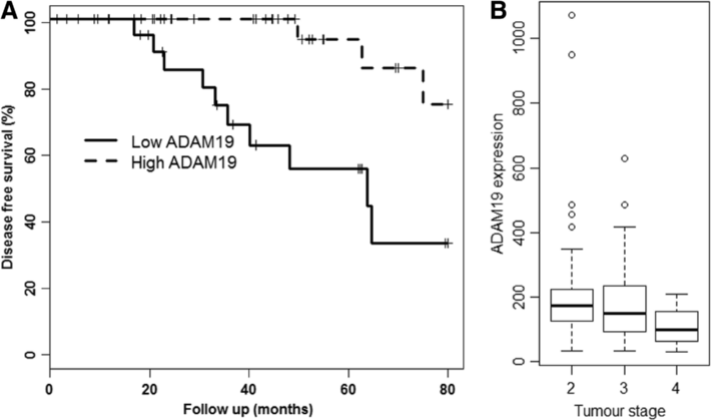
High ADAM19 expression correlates with increased disease-free survival and is associated with lower tumour stage. **a** Kaplan-Meier survival curve of the GSE40272 human prostate cancer cohort (*n* = 71). Relapse follow up is 80 months; *p* < 0.002. **b** TCGA prostate cancer boxplot of ADAM19 expression and tumour stage; *p* < 0.03; cor -0.179; *n* = 156

In order to evaluate the relationship between ADAM19 levels and prostate cancer, we studied ADAM19 expression in publicly available microarray data from two distinct cohorts of prostate cancer patients. In the GSE40272 cohort (Fig. 2a), there was a significant association between high median ADAM19 expression levels and reduced cancer relapse (Hazard Ratio 0.1749, *p* < 0.003). The clinicopathological characteristics of the GSE40272 cohort is presented in Table 1.

**Table 1:** Clinical information on the GSE40272 prostate cancer cohort

Patient feature	High ADAM19 (n)	Low ADAM19 (n)	Total (n)
All cases			77 (100 %)
ADAM19 evaluated	36 (50.7 %)	35 (49.3 %)	71 (92.2 %)
Age at diagnosis			
≤54	7 (43.8 %)	9 (56.3 %)	16 (22.5 %)
55-64	14 (45.2 %)	17 (54.8 %)	31 (43.7 %)
≥ 65	15 (62.5 %)	9 (37.5 %)	24 (33.8 %)
Cancer stage			
II	4 (80 %)	1 (20 %)	5 (7 %)
III	23 (46.9 %)	26 (53.1 %)	49 (69 %)
IV	7 (47.7 %)	8 (53.3 %)	15 (21.1 %)
Unknown	2 (100 %)	0 (0 %)	2 (2.8 %)
Gleason score			
6	7 (47.7 %)	8 (53.3 %)	15 (21.1 %)
7	26 (55.3 %)	21 (44.7 %)	47 (66.2 %)
8	1 (33.3 %)	2 (66.7 %)	3 (4.2 %)
9	1 (20 %)	4 (80 %)	5 (7 %)
Unknown	1 (100 %)	0 (0 %)	1 (1.4 %)
PSA levels (ng/mL)			
< 5	15 (45.5 %)	18 (54.5 %)	33 (46.5 %)
5-6.9	12 (80 %)	3 (20 %)	15 (21.1 %)
≥ 7	7 (35 %)	13 (65 %)	20 (28.2 %)
Unknown	2 (66.7 %)	1 (33.3 %)	3 (4.2 %)
Tumour recurrence			
Yes	5 (29.4 %)	12 (70.6 %)	17 (23.9 %)
No	29 (56.9 %)	22 (43.1 %)	51 (71.8 %)
Unknown	2 (66.7 %)	1 (33.3 %)	3 (4.2 %)

Clinicopathological features relative to ADAM19 mRNA expression levels in prostate tumours

In the TCGA cohort (Fig. 2b) we found that high ADAM19 expression in prostate cancer tissue was significantly negatively associated with tumour stage (cor = -0.18, *p* < 0.026). There were few deaths in the TCGA cohort, preventing any meaningful association of ADAM19 expression to overall survival. However, the TCGA cohort showed that high ADAM19 expression was highly associated with lower tumour stage, which taken together with the strong association of high ADAM19 expression with higher disease free survival, provides substantial evidence that ADAM19 is a marker of improved prognosis in prostate cancer.

### LNCaP cells proliferate at a faster rate than RWPE-1 cells

Having demonstrated that high levels of ADAM19 mRNA expression correlate with increased disease free survival and lower tumour stage in publicly available prostate cancer microarray databases, we then sought to determine the ADAM19 expression levels in human tumorigenic LNCaP prostate cancer cells and normal RWPE-1 prostate epithelial cells.

To ensure that our cells were displaying expected proliferative capacity, we conducted proliferation assays with LNCaP and RWPE-1 cells. As expected, we found that LNCaP cells proliferated significantly faster (*p* < 0.05) than normal RWPE-1 prostate epithelial cells at 1, 3 and 5 days post-seeding (Additional file 1: Figure S1).

### LNCaP cells express lower levels of ADAM19 compared to RWPE-1 cells

**Fig. 3:**
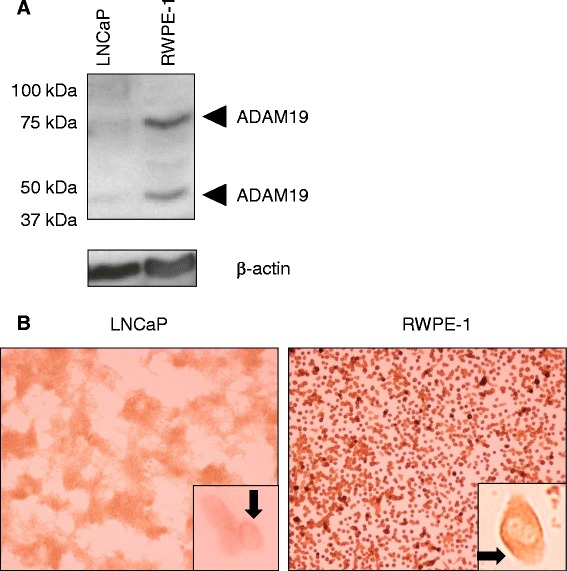
Human prostate cancer cells express lower levels of ADAM19 than normal prostate epithelial cells. **a** Western blotting for ADAM19 protein in androgen-sensitive human LNCaP cells and RWPE-1 cells. β-actin was used as a control. **b** ADAM19 protein expression in LNCaP and RWPE-1 cells as determined by immunocytochemistry. 100x magnification. Insert shows cytoplasmic staining

We next investigated the level of expression of ADAM19 protein in LNCaP cells and normal RWPE-1 cells. As shown in Fig. 3a, human ADAM19 is endogenously expressed in both LNCaP and RWPE-1 cells, but to different degrees. The 80 and 45 kD bands were observed at considerably higher levels in RWPE-1 cells, consistent with the notion that ADAM19 may act as a tumor suppressor in prostate cancer cells. Furthermore, RWPE-1 cells showed higher expression of endogenous ADAM19 protein in immunocytochemistry experiments (Fig. 3b). In addition, we found the intracellular expression of ADAM19 was cytoplasmic and heterogenous in both LNCaP and RWPE-1 cells. Taken together, these data suggest that normal prostate cells express significantly higher levels of ADAM19 compared to their tumorigenic counterparts.

### Verification that human ADAM19 is bioactive and cleaves human TNF-α

To determine the bioactivity of ADAM19 in our over-expression system, we conducted transfections in HEK293 cells. We showed that ADAM19 is readily over-expressed in HEK293 cells and that it is predominantly cytoplasmic and heterogeneous in distribution (Additional file 1: Figure S2A). Transfection efficiency, as determined using a GFP vector, was greater than 70 % (Additional file 1: Figure S3). Next we validated that we could use shedding of the pro-inflammatory cytokine TNF-α, a known substrate for ADAM19 [26, 27], as a bioassay for ADAM19 activity. Co-transfection of vectors expressing human TNF-α and human ADAM19 in HEK293 cells resulted in significantly increased TNF-α shedding (17-fold; *p* < 0.0001) (Additional file 1: Figure S2B). This confirmed in our system that ADAM19 can induce TNF-α cleavage, and generated a simple and reliable tool of its function. As PC3 prostate cancer cells are known to express TNF-α at the mRNA level, we also aimed to assess whether over-expression of ADAM19 in these cells may promote shedding of endogenous TNF-α. Unfortunately, no TNF-α protein was detected in cell-free culture supernatants after ADAM19 over-expression in PC3 cells (data not shown).

### Over-expression of human ADAM19 in LNCaP cells reduces proliferation

**Fig. 4:**
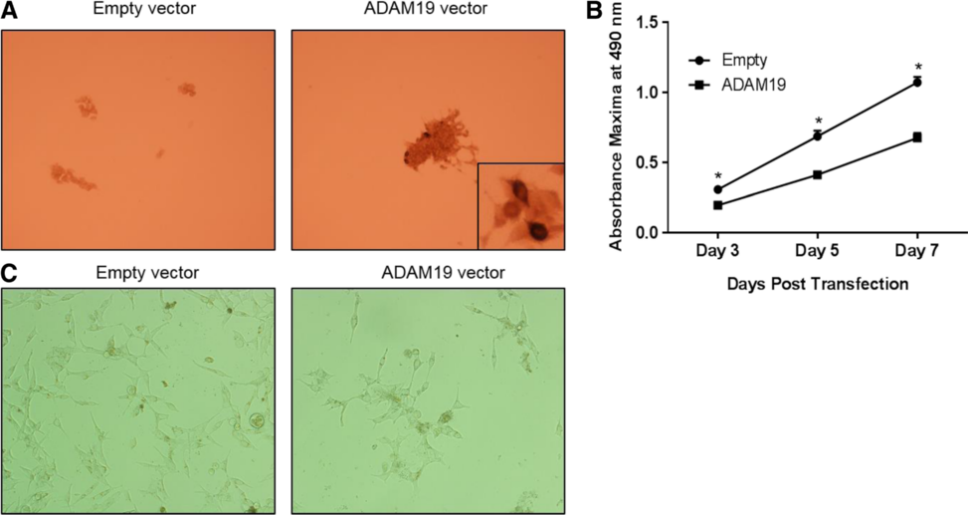
Human LNCaP cells are able to have ADAM19 over-expressed and ADAM19 over-expression reduces LNCaP cell proliferation. **a** LNCaP cells were transfected with either empty vector (pCR3.1) or human ADAM19 vector (pCR3.1 hADAM19) for 48 h before conducting ADAM19 immunocytochemistry. 100x magnification. Insert shows cytoplasmic staining of over-expressed ADAM19. **b** LNCaP cell proliferation was measured by MTS assay 3, 5, and 7 days after cells were transfected with empty vector or ADAM19 expressing vector. Mean + SEM. **c** Photomicrographs of LNCaP cells transfected with empty vector (pCR3.1) or ADAM19 expressing vector (pCR3.1 hADAM19) after 7 days post-transfection. 100x magnification; *n* = 12 samples/cell type/time point; *p < 0.05

We then aimed to ascertain the direct effects of ADAM19 over-expression on human prostate cancer cell proliferation. LNCaP cells were an ideal cell line for these studies, given their lower endogenous levels of ADAM19 (Fig. 3a and b). As shown in Fig. 4a, we were able to effectively overexpress ADAM19 in LNCaP cells, evidenced by strong cytoplasmic diaminobenzidine staining. Transfection efficiency, as assessed with a green fluorescent protein (GFP) expression vector, was more than 50 % (Additional file 1: Figure S4).

The effect of ADAM19 over-expression on LNCaP cell proliferation was determined by MTS assays performed 3, 5 and 7 days after transfection. Figure 4b shows that the proliferation rate of LNCaP cells overexpressing ADAM19 is significantly slower than LNCaP cells expressing empty vector (*p* < 0.05). Photomicrographs of LNCaP cells taken 7 days post-transfection (Fig. 4c) reinforce the LNCaP MTS proliferation assay results. Taken together, these data suggest that over-expression of human ADAM19 in LNCaP cells reduces proliferation.

### Over-expression of human ADAM19 in PC3 cells reduces migration and increases cell death

**Fig. 5:**
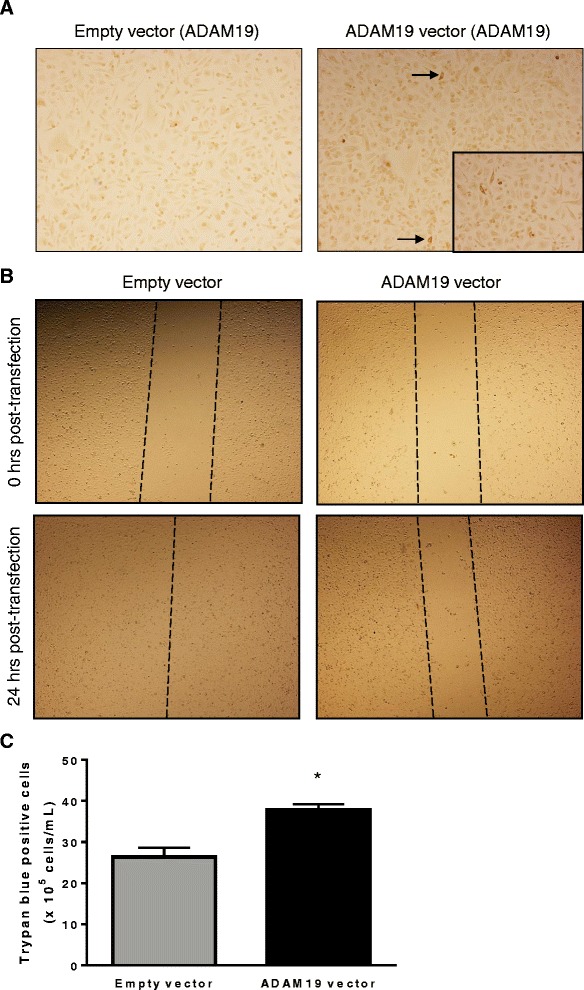
ADAM19 over-expression hinders human PC3 prostate cancer cell migration and increases death in PC3 cells. **a** PC3 cells were transfected with either empty vector (pCR3.1) or human ADAM19 vector (pCR3.1 hADAM19) for 48 h before conducting ADAM19 immunocytochemistry (100x magnification). Insert shows cytoplasmic staining of over-expressed ADAM19 (200x magnification). **b** Photomicrographs depict PC3 cell migration 0 and 24 h post-transfection with either empty vector or human ADAM19 vector (40x magnification). Images are representative of 4 individual wells. **c** A trypan blue count was conducted on empty vector or human ADAM19 vector transfected cells after 48 h of transfection; *p < 0.005; *n* = 4

Lastly, we examined the impact of ADAM19 over-expression on human prostate cancer cell migration. Androgen-independent PC3 cells were utilised because of their low endogenous expression of ADAM19 (Fig. 5a) and ability to consistently grow in a monolayer. The effect of ADAM19 over-expression on PC3 cell migration was evaluated with an in vitro scratch assay conducted on transfected PC3 cells. We show for the first time that ADAM19 over-expression hinders migration of PC3 cells reproducibly compared with cells transfected with empty vector, 24 h post-transfection (Fig. 5b). This migration pattern was also observed 48 h after the initial transfection (data not shown). In addition, we assessed the cellular viability of PC3 cells 48 h after transfection. We found that ADAM19 transfected PC3 cells experienced statistically significant higher cell death than empty vector transfected cells (Fig. 5c) which is a novel discovery.

## Discussion

We have shown for the first time that ADAM19 may serve as a tumor suppressor in human prostate cancer. Our examination of microarray data from two independent human prostate cancer cohorts indicated that high ADAM19 expression was associated with almost a six-fold increase in disease free survival and a significantly lower tumour stage. These data prompted us to further investigate the direct effect of ADAM19 in human prostate cancer cells. We found that ADAM19 expression is reduced in human prostate cancer cells compared to normal prostate epithelial cells.

Interestingly and in contrast to our findings herein, previous studies have demonstrated that higher ADAM19 expression may be pro-oncogenic and is considered to play a role in driving development of human ovarian and renal cancer [28, 29] and increased expression is associated with human brain tumour invasiveness [30]. Thus, although ADAM19 appears to be involved in driving other cancers, it appears to have the opposite effect in human prostate cancer. Over-expression of human ADAM19 in LNCaP or PC3 cells reduced the proliferation rate and migration of these cells respectively. Collectively, these findings suggest that ADAM19 is a beneficial factor in prostate cancer and functions by decreasing proliferation and migration.

Although our study suggests that ADAM19 is a tumour suppressor in prostate cancer, the mechanism is still to be elucidated. There are numerous potential candidate proteins which are known to be either substrates or binding proteins of ADAM19. Examples of substrates include Neuregulin 1-β1 [31], TNF-α [26, 27, 32], and cysteine-rich protein 2 (CRIP-2) [33]. The binding proteins include α4β1 and α5β1 integrins [20]. ADAM19’s metalloproteinase domain is involved in the catalytically-mediated ectodomain shedding of substrates [15]. One substrate cleaved by ADAM19 is Neuregulin 1-β1 [31]. This substrate has been identified to bind to the tyrosine kinase receptors ErbB3 and ErbB4 to result in tyrosine residue phosphorylation. This ultimately affects cardiac development and morphogenesis [34–36]. Grasso et al*.* (1997) [37] demonstrated that Neuregulin binding to ErbB3 and ErbB4 ligands inhibited LNCaP growth. In mice, the shedding of Neuregulin 1-β1 appeared to be enhanced by ADAM19’s transmembrane domain [38]. The cleavage of Neuregulin 1-β1 by ADAM19 may therefore signify a possible anti-tumourigenic mechanism in human prostate cancer.

The pro-inflammatory cytokine TNF-α is another substrate that ADAM19 has been shown to cleave in a variety of settings [26, 27, 32]. Chopra et al*.* (2004) [39] determined that LNCaP cells are sensitive to TNF-α stimulated growth arrest and apoptosis. TNF-α has been shown to induce apoptosis in human prostate cancer cell lines mainly through the NFκB pathway, however, it appears that this may be partly dependent upon androgen sensitivity [39, 40]. Our data indicates that ADAM19 induces TNF-α shedding in HEK293 cells, and further studies are required to elucidate the effect of TNF-α shedding by ADAM19 and it’s role in human prostate cancer. We are aware from our current study, that ADAM19 appears not to be shedding endogenous TNF-α from PC3 prostate cancer cells.

The cysteine-rich domain enables ADAM19 to possess autolytic processing activity. ADAM19’s cysteine-rich and disintegrin domains associate with CRIP2 to result in the release of CRIP-2 [33]. This tumour-suppressor protein reduces tumourigenesis and angiogenesis in nasopharyngeal cancer cell lines and tumours [41]. CRIP-2 also promotes apoptosis of esophageal cancer cells [42]. Importantly, CRIP-2 is known to be expressed by LNCaP cells [43] as well as normal prostate epithelial cells [44], however the expression differences between normal and cancerous prostate tissue in humans remains to be explored. ADAM19’s ability to increase secretion of CRIP-2 may represent another possible anti-tumourigenic mechanism for ADAM19 in prostate cancer [33].

It has been shown that ADAM19 inhibits migration mediated by the α4β1 and α5β1 integrins by binding to these integrins with its disintegrin domain [20]. Hence, ADAM19 neutralises the actions of α4β1 and α5β1 integrins. The integrin α4β1 normally binds to fibronectin, causing growth factor and tumour-induced lymphangiogenesis [21]. Integrin α5β1 usually mediates fibronectin adhesion necessary for prostate cancer metastasis [45]. Excitingly, neutralisation of the activity of α4β1 and α5β1 by ADAM19 may be a mechanism by which this metalloproteinase reduces the progression of prostate cancer. Interestingly, we have also shown that over-expression of ADAM19 in PC3 human prostate carcinoma cells inhibits migration of these cells. Future studies will address whether this is mediated by interaction of ADAM19 with integrins.

It is interesting to speculate regarding the post-translational modifications that may be at play in prostate cancer and may contribute to the reduced ADAM19 expression in prostate cancer. Further studies should aim to assess whether the ADAM19 gene is hypermethylated and silenced in prostate cancer [46].

An exciting avenue for future investigation is the study of single nucleotide polymorphisms (SNPs) within *ADAM19* in human prostate cancer. London et al*.* [47] identified that a nonsynonymous serine to glycine substitution within *ADAM19* (rs1422795) could affect human pulmonary function. The functional relevance of rs1422795 on ADAM19 expression is currently unknown. It will be of interest to assess if there are any polymorphisms that reduce human ADAM19 expression, particularly in the prostate cancer environment.

## Conclusions

Our study provides evidence that elevated ADAM19 expression may serve as a tumor suppressor in human prostate cancer. Using human normal and prostate cancer biopsies, we show that ADAM19 protein levels are elevated in normal prostate and reduced in prostate cancer specimens. Our clinical data from two different cohorts provides compelling results for involvement of ADAM19 in prostate cancer and our in vitro data shows that ADAM19 can regulate prostate cancer cell growth and migration. Intriguingly, it would appear that the effects of ADAM19 may be limited to prostate cancer cells with reduced expression of ADAM19. These data provide for the first time a foundation to further explore the mechanism by which ADAM19 exerts its tumor suppressor effects and raises the possibility that expression of ADAM19 levels in prostate cancer tumors could become a useful biomarker in the disease.

## Additional file

Additional file 1:
**Figure S1.** Human prostate cancer cells proliferate faster than normal prostate epithelial cells. Cell proliferation was measured by MTS assay 1, 3 and 5 days after cells were seeded (0.25x10^5^ cells/mL on day 0); *n* = 12 samples/cell type/time point; mean +/- SEM; **p* < 0.05. **Figure S2.** Over-expression of human TNF-α with human ADAM19 in HEK293 cells promotes TNF-α shedding. (A) HEK293 cells were transfected with either empty vector (pCR3.1) or human ADAM19 vector (pCR3.1 hADAM19). Insert shows cytoplasmic staining of over-expressed ADAM19. 100x magnification. (B) Comparison of HEK293 cells transfected with ADAM19 expression vector alone, TNF-α vector alone or co-transfected with both vectors; mean +/- SEM; **p* = 0.0004; ***p* < 0.0001.** Figure S3.** Transfection efficiency in HEK293 cells 48 h post-transfection. Phase contrast (A) and green fluorescent protein (GFP) positivity (B). 100x magnification. **Figure S4.** Transfection efficiency in LNCaP cells 48 h post-transfection. Phase contrast (A) and green fluorescent protein (GFP) positivity (B). 100x magnification. (DOCX 1817 kb)

## Abbreviations

ADAM: A Disintegrin and Metalloproteinase 19
BPE: bovine pituitary extract
CRIP-2: cysteine-rich protein 2
DAB: diaminobenzidene
DMEM: Dulbecco’s Modified Eagle Medium
EGF: epidermal growth factor
GEO: Gene Expression Omnibus
GFP: green fluorescent protein
HEK293: human embryonic kidney cells
HRP: horse-radish peroxidase
IGF-1: insulin-like growth factor 1
K-SFM: keratinocyte serum free medium
MMP: matrix metalloproteinases
RPMI: roswell park memorial institute
TCGA: the cancer genome atlas
SDS: sodium dodecyl sulfate
TNF-α: tumor necrosis factor-α
