# Supplementary material for: Genetic and cellular studies highlight that A Disintegrin and Metalloproteinase 19 is a protective biomarker in human prostate cancer
Source: BMC Cancer. 2016 Feb 24;16:151. doi: 10.1186/s12885-016-2178-4 (PMC4766641; doi:10.1186/s12885-016-2178-4)
Supplement: Additional file 1: — Figure S1. Human prostate cancer cells proliferate faster than normal prostate epithelial cells. Cell proliferation was measured by MTS assay 1, 3 and 5 days after cells were seeded (0.25x10^5^ cells/mL on day 0); n = 12 samples/cell type/time point; mean +/- SEM; *p < 0.05. Figure S2. Over-expression of human TNF-α with human ADAM19 in HEK293 cells promotes TNF-α shedding. (A) HEK293 cells were transfected with either empty vector (pCR3.1) or human ADAM19 vector (pCR3.1 hADAM19). Insert shows cytoplasmic staining of over-expressed ADAM19. 100x magnification. (B) Comparison of HEK293 cells transfected with ADAM19 expression vector alone, TNF-α vector alone or co-transfected with both vectors; mean +/- SEM; *p = 0.0004; **p < 0.0001. Figure S3. Transfection efficiency in HEK293 cells 48 h post-transfection. Phase contrast (A) and green fluorescent protein (GFP) positivity (B). 100x magnification. Figure S4. Transfection efficiency in LNCaP cells 48 h post-transfection. Phase contrast (A) and green fluorescent protein (GFP) positivity (B). 100x magnification. (DOCX 1817 kb) [file 12885_2016_2178_MOESM1_ESM.docx]

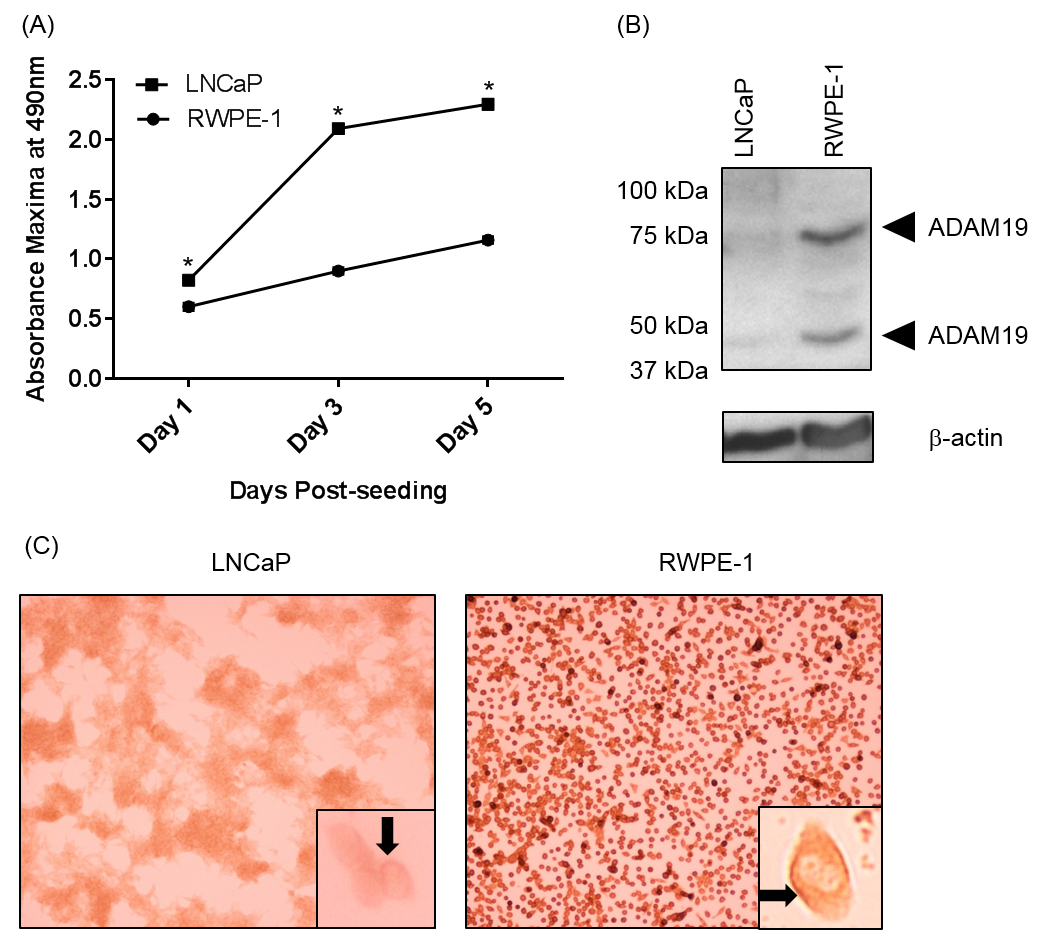


**Supplementary Figure 1. Human prostate cancer cells proliferate faster than normal prostate epithelial cells.** Cell proliferation was measured by MTS assay 1, 3 and 5 days after cells were seeded (0.25x10^5^ cells/mL on day 0); n = 12 samples/cell type/time point; mean + SEM; *p < 0.05.


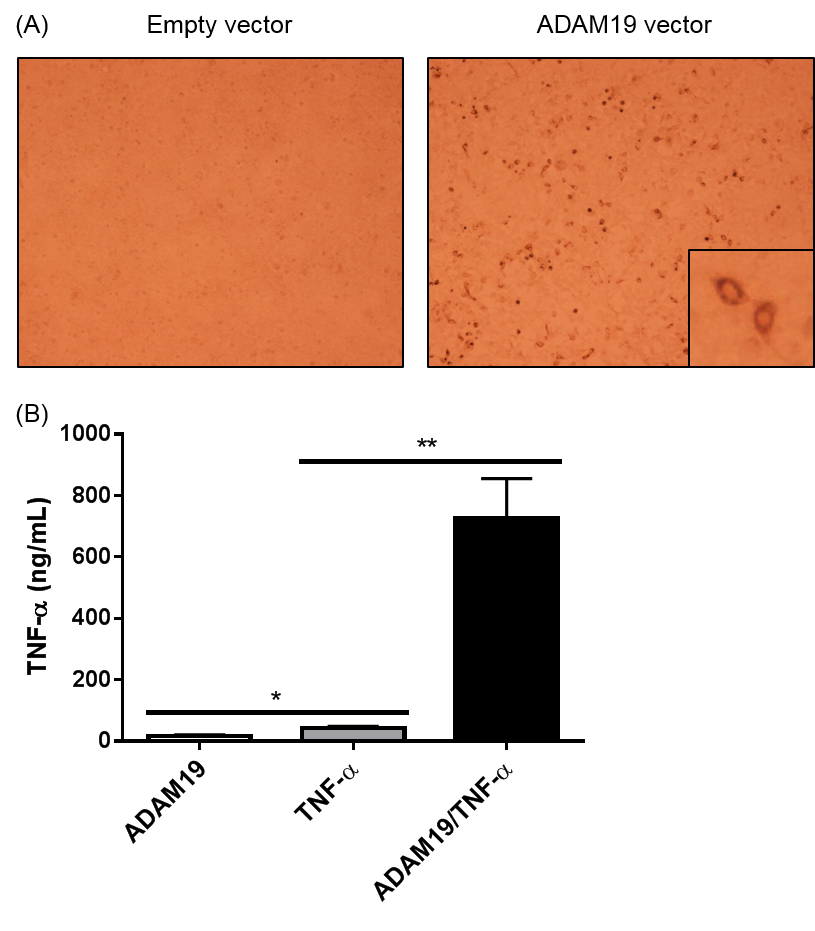


**Supplementary Figure 2. Over-expression of human TNF-α with human ADAM19 in HEK293 cells promotes TNF-α shedding.**  (A) HEK293 cells were transfected with either empty vector (pCR3.1) or human ADAM19 vector (pCR3.1 hADAM19). Insert shows cytoplasmic staining of over-expressed ADAM19. 100x magnification. (B) Comparison of HEK293 cells transfected with ADAM19 expression vector alone, TNF-α vector alone or co-transfected with both vectors; mean + SEM; *p = 0.0004; **p < 0.0001.

**
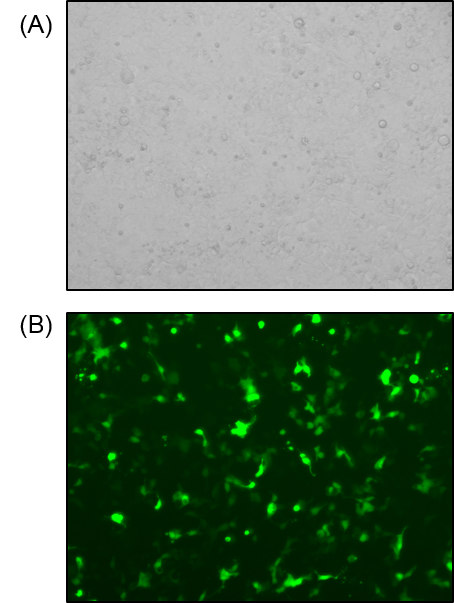
**

**Supplementary Figure 3. Transfection efficiency in HEK293 cells 48 hours post-transfection.** Phase contrast (A) and green fluorescent protein (GFP) positivity (B). 100x magnification.


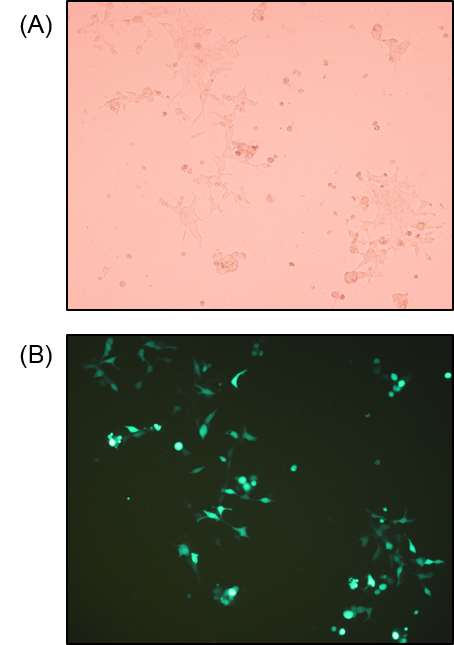


**Supplementary Figure 4. Transfection efficiency in LNCaP cells 48 hours post-transfection.** Phase contrast (A) and green fluorescent protein (GFP) positivity (B). 100x magnification.
